# (CONSORT) Wound closure using Dermabond after excision of hemangioma on the lip

**DOI:** 10.1097/MD.0000000000015342

**Published:** 2019-04-26

**Authors:** Jung Woo Chang, Kyu Sang Cho, Woong Heo, Jang Hyun Lee

**Affiliations:** Department of Plastic and Reconstructive Surgery, Hanyang University Guri Hospital, Hanyang University College of Medicine, Guri, South Korea.

**Keywords:** Dermabond, hemangioma, lip

## Abstract

**Background:**

As the lip contains ample blood supply, hemangiomas often occur in this area. When surgical excision is performed, wound closure is important. To prevent infection from saliva and food, watertight wound closure is needed. The purpose of this study is to demonstrate the usefulness of Dermabond for wound closure after hemangioma excision on the lip.

**Methods:**

Between December 2015 and August 2017, 11 patients with lip hemangioma underwent surgical excision. When closing the wound, Dermabond was used for skin closure after subcutaneous sutures. Demographic data and complications were recorded. Scars were evaluated with the Vancouver scar scale (VSS), and the postoperative shape of the lip was assessed on a 10-point satisfaction scale at 1 month and 6 months postoperatively.

**Results:**

All cases completely healed without any complications, such as wound dehiscence or infection. There were no recurrences at postoperative 1 month during the follow-up period. The aesthetic results of the scars were also excellent. The average VSS score on postoperative 1 month was 4.2, and it decreased to 2.2 at postoperative 6 months. The average patient satisfaction score at postoperative 1 month was 7.4, and it increased to 9.5 at postoperative 6 months.

**Conclusion:**

Dermabond is useful for wound closure after hemangioma excision on the lip. It prevents wound contamination, and yields acceptable aesthetic results.

## 1 Introduction

Hemangioma is one of the most common benign tumors of vascular origin, and it usually occurs on the skin, lips, or inside the mouth as a painless, red-to-blue colored lesion.^[1]^ The lip is a common site of hemangioma formation, as it contains ample blood supply. When hemangioma occurs on the lip, it can distort the lip anatomy as it grows, with concomitant risks of ulceration, bleeding, scarring, and contour deformity.^[2]^

The treatment of lip hemangioma depends on the size, location, and stage of the lesion.^[1]^ Many different procedures, such as embolization, steroid therapy, cryosurgery, and electrodessication have been used depending on the condition of the lesion. However, surgical excision cannot be avoided if a hemangioma of the lip grows like a mass, which makes patients uncomfortable and worried.^[3]^ Dye lasers or neodymium-doped yttrium aluminum garnet (Nd:YAG) lasers can also be successfully used for lesions that show excessive growth,^[4–6]^ but laser treatment takes a long time and requires several visits to an outpatient clinic. In contrast, hemangioma can be completely removed by surgical excision in a short time.^[3]^ When surgical excision is performed on a hemangioma of the lip, wound closure is important. To prevent infection from saliva and food, watertight wound closure is needed. Furthermore, minimizing the scar is important, as lip scars are distinctive from scars in other locations.^[7]^

To obtain satisfactory functional and aesthetic outcomes, Dermabond, which is made of octyl-2-cyanoacrylate, can be used to provide a barrier to dirt and for scar management.^[8]^ The purpose of this study is to demonstrate the usefulness of Dermabond for wound closure after hemangioma excision on the lip.

## 2 Methods

This study was conducted in conformity with the World Medical Association Declaration of Helsinki, and the protocol was approved by the Institutional Review Board of Hanyang University Guri Hospital in July, 2018 (2018-07-007-002) and written informed consents were obtained the patients for publication of cases. Between December 2015 and August 2017, 11 patients with lip hemangioma underwent surgical excision. All patients eligible for study inclusion had a lip hemangioma <2 cm in diameter and the area of the lesion was limited to within the vermilion of the lip. We exclude a lip hemangioma >2 cm because we could not repair completely with Dermabond. Patients who were excluded from study enrollment were those with Dermabond closure on the lip outside vermilion because many papers were published for Dermabond closure on ordinary skin.

Under local anesthesia, patients underwent complete excision with fine dissection. After bleeding control with electrocauterization, subcutaneous sutures were made with absorbable material (Vicryl 6-0, Ethicon, Inc., Somerville, NJ). When the subcutaneous sutures were made, manual compression was performed with gauze for few seconds for dermal bleeding control. After confirming that the wound was clean without a blood clot, Dermabond (Ethicon, Inc., Somerville, NJ) was painted over the wound edge. The wound was then held in place for 30 seconds for complete polymerization. As the wound became waterproof, patients could eat and drink without any restrictions. The Dermabond was removed on postoperative day 7 in the outpatient clinic. Before postoperative day 7, patients did not visit the clinic unless the Dermabond had become detached.

A retrospective review of the case notes was performed. Data on biopsy results, defect size, and complications were collected. Aesthetic results such as scar status and the shape of the lip were evaluated at 1 month and 6 months postoperatively. Scars were evaluated using the Vancouver Scar Scale (VSS). The postoperative shape of the lip was assessed using a patient satisfaction questionnaire, on which a score of 10 points corresponded to the highest level of satisfaction.

## 3 Results

The mean age of the patients was 52.1 years (range, 17–80 years). The defect size ranged from 0.64 to 2.25 cm^2^. All 11 cases were cavernous hemangioma, as confirmed by biopsy. Primary wound closure with Dermabond after complete excision was possible in all cases by a single surgeon (JHL). There were no complications, such as wound dehiscence or infection, in any case. There were no recurrences during the follow-up period. Dermabond was maintained until complete wound healing in 10 cases. Only 1 patient experienced Dermabond detachment within 7 days postoperatively, which led to a re-touch procedure of Dermabond painting (Table 1).

**Table 1:**
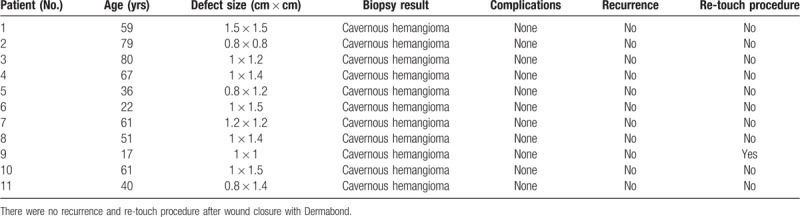
Demographic, defect size, biopsy, and complications of the patients.

Scar status improved during the follow-up period, as indicated by decreasing VSS scores. The average VSS score at postoperative 1 month was 4.2, and it decreased to 2.2 at postoperative 6 months. The patients’ satisfaction with their postoperative lip shape increased during the follow-up period. The average satisfaction score at postoperative 1 month was 7.4, and it increased to 9.5 at postoperative 6 months. There were no cases of lip deformity during this period (Table 2).

**Table 2:**
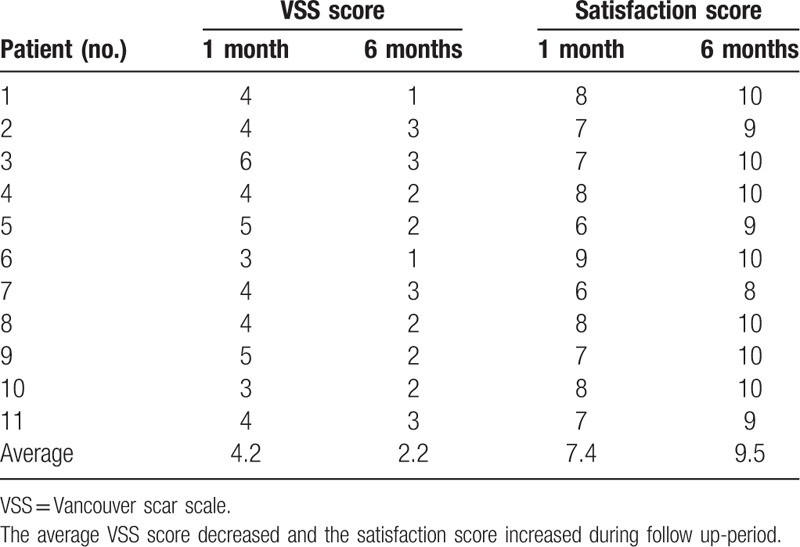
VSS score and patients’ satisfaction score for lip wounds.

### 3.1 Case 1

**Figure 1:**
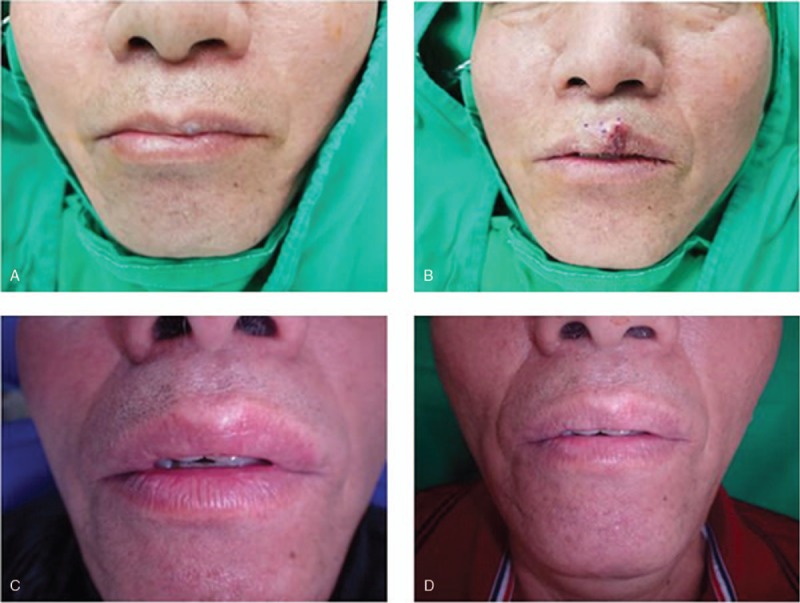
A 61-year-old male patient who underwent hemangioma excision on the lip. Dermabond was applied on the surgical wound, and the wound healed without any complications. Preoperative (A), immediately postoperative (B), 1-month postoperative (C), and 6-month postoperative (D) photographs were taken.

A 61-year-old male patient with a lip hemangioma underwent surgical excision (Fig. 1). The mass measured 1 × 1.5 cm, and it was confirmed to be a cavernous hemangioma. Dermabond was applied on the surgical wound, and the wound healed without any complications or a re-touch procedure. The wound scar improved, as the VSS score decreased from 3 at 1 month postoperatively to 2 at 6 months. The satisfaction score increased from 8 at 1 month postoperatively to 10 at 6 months.

### 3.2 Case 2

**Figure 2:**
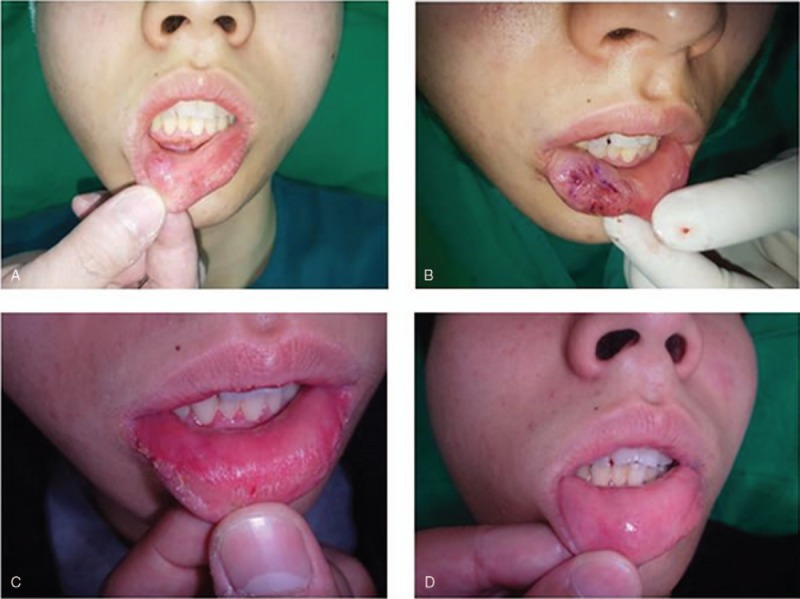
A 17-year-old male patient who underwent hemangioma excision on the lip. Dermabond was applied for the wound closure, and the wound healed without any complications. Preoperative (A), immediately postoperative (B), 1-month postoperative (C), and 6-month postoperative and (D) photographs were taken.

This case involved a 17-year-old male patient who underwent surgical excision (Fig. 2). The mass measured 1 × 1 cm, and it was confirmed to be a cavernous hemangioma. After complete excision, Dermabond was applied for wound closure. The patient experienced Dermabond detachment at postoperative 3 days, and a re-touch procedure was performed in the outpatient clinic. There was no problem with wound healing, which proceeded without any complications. The VSS score for the scar was 5 at 1 month postoperatively and 2 at 6 months, respectively. The satisfaction score at 1 month postoperatively was 7, and it increased to 10 at 6 months postoperatively.

## 4 Discussion

Cyanoacrylates were first synthesized in 1949 and have been used clinically since 1959. The chemical structure of these compounds was developed with the goal of creating effective adhesives that are safe and easy to use,^[8–10]^ and currently Dermabond (octyl-2-cyanoacrylate) is a widely used topical skin adhesive after being approved by the U.S. Food and Drug Administration in 1998.^[8]^ As it has high tensile strength and many advantages, it has been a good alternative to wound closure using sutures due to its safety, effectiveness, and excellent cosmetic outcomes.^[11–13]^

Dermabond is a liquid monomer. When it comes into contact with tissue surfaces, it polymerizes to form a strong flexible film on the edges of a wound.^[8]^ Upon application, partially ionized water molecules on the skin surface neutralize the stabilizer, thereby allowing polymerization to occur usually within 10 seconds.^[14–16]^ Regarding healing potential, wound strength, and cosmesis, Dermabond has been shown to be equivalent to sutured skin closure.^[15,17]^ When Dermabond is applied on a wound, the moist occluded environment helps wound healing. It acts as a barrier against bacterial colonization and prevents the ingress of microbes from the surrounding environment to the wound.^[18–20]^

Dermabond has been used in a wide variety of clinical settings.^[21–23]^ Because it does not require suture removal, it is especially widely used in pediatric surgery, including for skin closure in cleft lip procedures and circumcision.^[21,24,25]^ For cleft lip surgery, Dermabond provides benefits including spontaneous peel-off, good tensile strength, microbial barrier properties, a shorter application time than sutures, and the fact that it allows contact with water immediately after surgery.^[21]^ For the same reasons, it is beneficial to apply Dermabond to adults for wound closure after hemangioma excision on the lip.

Care should be taken when a surgical wound is made on the lip, as passage of saliva, water, and various foods can cause wound contamination. When the conventional suture technique is used, dirty liquid can run into the intervals of the suture lines. The application of Dermabond over the edges of a wound that is closed only by subcutaneous sutures can protect the wound from the unsterile environment. It has been proved that octyl-2-cyanoacrylate forms a microbial barrier that successfully prevents the infiltration of skin and oral cavity microflora.^[20]^ Patients can eat, drink, shower, and brush their teeth without the possibility of accidental stitch-out caused by a toothbrush. They do not need to gargle or apply an ointment or dressing. These factors make it easy to treat patients, with low cost and a short treatment time. As no patients in this study experienced any complications, this method can be applied effectively. Although 1 patient experienced Dermabond detachment before postoperative day 7, and a re-touch procedure was performed, there was no problem with safe wound healing. As the 10 other patients showed satisfactory maintenance of Dermabond for 7 days, it can be concluded that Dermabond provided appropriate tensile strength during the wound healing period. If a patient experiences Dermabond detachment, safe wound healing can continue after an appropriate re-touch procedure of Dermabond painting. Dermabond, itself, is 2 times more expensive than normal suture materials. However, using Dermabond reduces number of outpatient clinic visiting and this brings a cost benefit to patient's total pay.

Postoperative scarring and shape deformity of the lip are another important issue. As the lip is an organ with unique shape, even a small distortion of its shape by scarring can be seen in a magnified form. Furthermore, repetitive movements of the lip increase the risk of scar hypertrophy.^[26–28]^ Every case in this study showed an acceptable VSS score during the follow-up period. The scar status improved during the follow-up period, and the final average VSS score at postoperative 6 months was 2.2, indicating that the structural shape of the lip had not been distorted. The patients’ satisfaction score with the postoperative lip shape increased during the follow-up period. The final average score at postoperative 6 months was 9.5, which is close to full marks. These results prove that using Dermabond for lip wound closure provided aesthetically acceptable outcomes.

Unfortunately, the absence of a control group in which conventional simple sutures were used is a limitation of this study. The small sample size is second drawback of this study. This is a preliminary study because of its small sample size, and more studies with larger groups are needed. However, the authors suggest that our study provides meaningful evidence of the efficacy of Dermabond on lip wounds. We evaluated lip scar by VSS and patient satisfaction score with only internal evaluators and patients. For the more objective evaluation, the patient and observer scar assessment scale (POSAS) with 3 variables including internal and external evaluators and patients should be included. This is third limitation of this study.

Several studies have investigated allergic contact dermatitis after using Dermabond for wound repair.^[29–31]^ However, none of the patients in this study experienced contact dermatitis. This may have been because the lip is a relatively hydric organ, as arid environments contribute to contact dermatitis. When Dermabond is applied on the lip, patients can avoid an excessively dry environment after surgery. However, the mechanism of how patients can avoid contact dermatitis is still unclear, and a further study with a larger number of cases is needed to elucidate the mechanism.

## 5 Conclusion

The authors propose that Dermabond could be a safe and effective tool for wound closure after hemangioma excision on the lip. It can prevent visible scar formation and wound contamination from saliva and food residue. It is convenient for patients because there is no requirement for them to visit an outpatient clinic for dressing and stitch-out.

## Author contributions

**Conceptualization:** Jung Woo Chang, Jang Hyun Lee.

**Data curation:** Jung Woo Chang, Kyu Sang Cho, Woong Heo, Jang Hyun Lee.

**Formal analysis:** Kyu Sang Cho, Woong Heo, Jang Hyun Lee.

**Investigation:** Jung Woo Chang, Kyu Sang Cho, Woong Heo.

**Methodology:** Jung Woo Chang, Jang Hyun Lee.

**Project administration:** Woong Heo.

**Resources:** Kyu Sang Cho, Jang Hyun Lee.

**Supervision:** Jung Woo Chang, Jang Hyun Lee.

**Writing – original draft:** Jung Woo Chang.

**Writing – review & editing:** Jung Woo Chang.
